# Appointment of Dr. S. D. Gross

**Published:** 1859-10

**Authors:** 


Dr. S. D. Gross has been appointed to fill the vacancy caused by the resignation of Dr. R. L. Madison, in the Surgical Department of the Howard Hospital, Philadelphia.

